# Fatty acid signatures connect thiamine deficiency with the diet of the Atlantic salmon (*Salmo salar*) feeding in the Baltic Sea

**DOI:** 10.1007/s00227-018-3418-8

**Published:** 2018-10-01

**Authors:** Marja Keinänen, Reijo Käkelä, Tiina Ritvanen, Jukka Pönni, Hannu Harjunpää, Timo Myllylä, Pekka J. Vuorinen

**Affiliations:** 1 Natural Resources Institute Finland (Luke), P.O. Box 2, FI-00791 Helsinki, Finland; 2 Department of Biosciences, University of Helsinki, P.O. Box 65, FI-00014 Helsinki, Finland; 3 Finnish Food Safety Authority Evira, Mustialankatu 3, FI-00790 Helsinki, Finland; 4 Natural Resources Institute Finland (Luke), Puuvillakuja 6, FI-65200 Vaasa, Finland; 5 Natural Resources Institute Finland (Luke), Itäinen Pitkäkatu 4 a, FI-20520 Turku, Finland

## Abstract

Thiamine (vitamin B1) deficiency in salmonids related to a lipid-rich fish diet causes offspring mortality in the yolk-sac fry phase. A low free thiamine (THIAM) concentration in eggs is an indication of this syndrome. Thiamine deficiency of salmon (*Salmo salar*) feeding in the Baltic Sea, called M74, was connected to the principal prey fish and feeding area using fatty acid (FA) signature analysis. The FAs of feeding salmon from two areas of the Baltic Sea, the Baltic Proper (57°10′ 19°30′) and the Bothnian Sea (61°30′ 20°00′) in 2004, reflected the principal prey species in these areas, sprat (*Sprattus sprattus*) and herring (*Clupea harengus*), respectively. Arachidonic acid (ARA, 20:4n-6) and 18:1n-7 indicated dietary herring, 18:1n-9 dietary sprat and 14:0 feeding in the Baltic Proper. The muscle FA profile of non-M74 female spawners of the River Simojoki in a year (1998) with a moderate M74 incidence and salmon of a non-M74 year (2004) reflected herring FAs, whereas the FAs in the M74 year and specifically in M74 females displayed characteristics of sprat. In the M74 year, the THIAM concentration had the strongest positive correlation with the proportion of muscle ARA, and the strongest negative correlations with 14:0 and the ratios 18:1n-9/ARA and 14:0/ARA. Thus, ARA along with 14:0 and these ratios were the most sensitive FA indicators of the dietary species and origin of the M74 syndrome. Despite the pre-spawning fasting, tissue FA signatures were consequently able to connect dietary sprat in the Baltic Proper with thiamine deficiency in Baltic salmon.

**Electronic supplementary material:**

The online version of this article (10.1007/s00227-018-3418-8) contains supplementary material, which is available to authorized users.

## Introduction

**Fig. 1:**
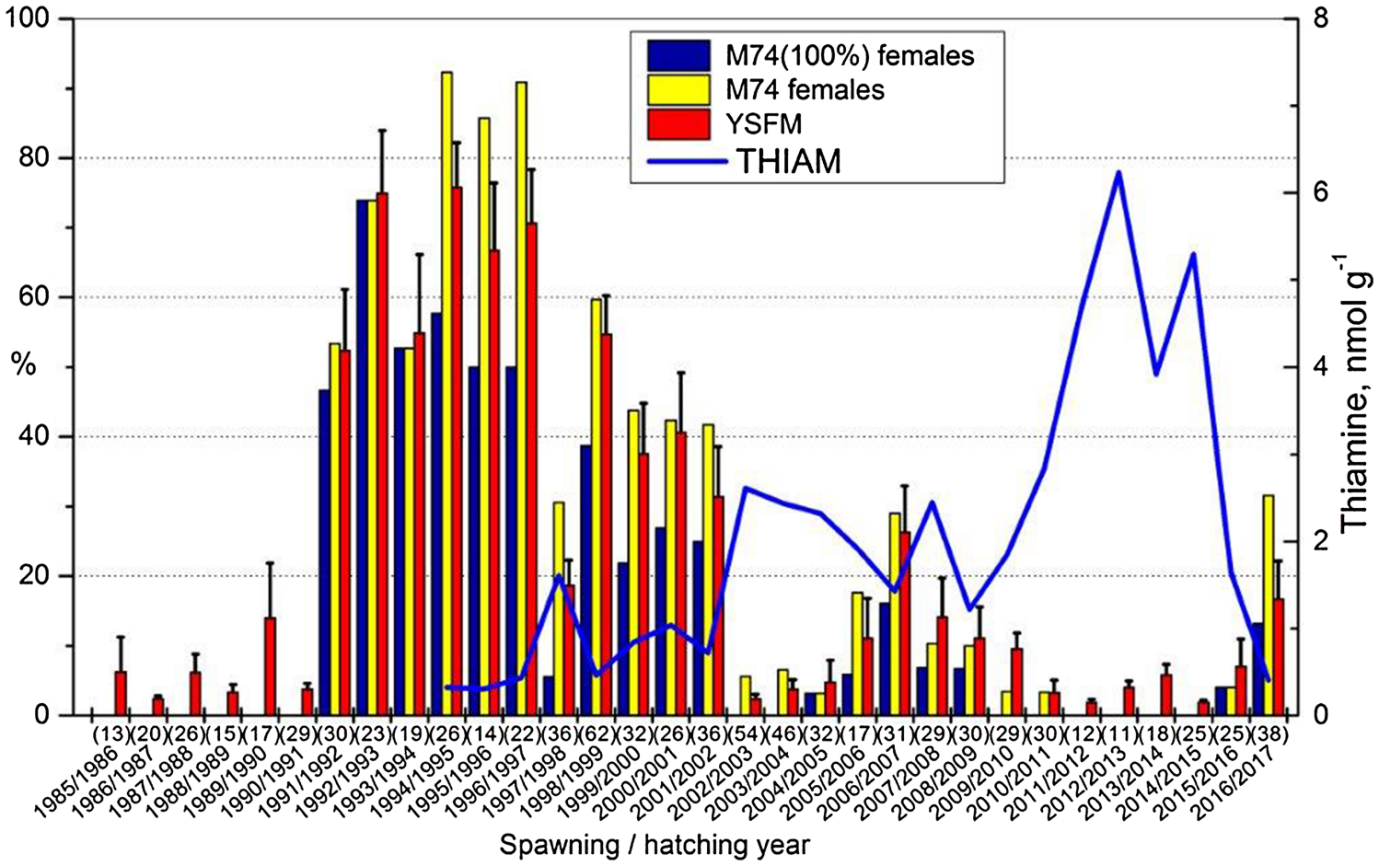
Annual (spawning year/hatching year) proportions of those Baltic salmon females (total number of sampled females in parentheses) ascending the River Simojoki whose offspring have all died of thiamine deficiency [M74 (100%) females] or whose offspring during the yolk-sac fry phase have displayed symptoms and mortality related to thiamine deficiency (M74 females), and the mean (± SE) annual yolk-sac fry mortality (YSFM), with a line representing the annual median free thiamine concentration (THIAM) of unfertilized eggs

Thiamine (vitamin B1) deficiency disturbing the reproduction of Atlantic salmon (*Salmo salar*) feeding in the Baltic Sea (hereafter salmon or Baltic salmon) is known as the M74 syndrome (Bengtsson et al. 1999). M74 has been connected to a lipid-rich diet, and specifically one including young sprat (*Sprattus sprattus*) with a high lipid content (Karlsson et al. 1999a; Mikkonen et al. 2011; Keinänen et al. 2012). Another important prey fish of Baltic salmon is the herring (*Clupea harengus*), of which salmon prefer smaller specimens (Hansson et al. 2001; Vuorinen et al. 2014a). Thiamine deficiency manifests in salmon yolk-sac fry [i.e., eleutheroembryos or free embryos (Balon 1975)] as symptoms and mortality (Bengtsson et al. 1999; Keinänen et al. 2000), which increase when the thiamine concentration in eggs is very low (Fig. 1) (Amcoff et al. 1999; Vuorinen and Keinänen 1999; Koski et al. 2001). Among brood salmon caught and kept for egg stripping, wriggling behaviour and mortalities have also been recorded (Bengtsson et al. 1999; Keinänen et al. 2014). The concentration of astaxanthin and the total concentration of carotenoids are also on average lower in thiamine-deficient eggs (Lundström et al. 1999; Vuorinen and Keinänen 1999; Keinänen et al. 2014), suggesting a connection with oxidative stress (Pickova et al. 1998; Lundström et al. 1999; Pickova et al. 2003). The death of yolk-sac fry, however, results from the depletion of thiamine reserves of the yolk (Bylund and Lerche 1995; Amcoff et al. 1999; Koski et al. 1999; Vuorinen and Keinänen 1999).

Thiamine is an essential micronutrient, which has a central role in energy metabolism (Lonsdale 2006) and also a linkage to fatty acid (FA) metabolism. Moreover, thiamine serves as an antioxidant (Lukienko et al. 2000; Gibson and Zhang 2002). Fish need to obtain thiamine from their diet (Niimi et al. 1997), and the requirement for it depends on the energy density of the food (Woodward 1994). As the net energy value of lipids is more than double that of proteins (Kriketos et al. 2000), the need for thiamine largely depends on the lipid content of prey fish.

In addition to sprat containing more lipid overall than herring, the youngest sprat have the highest lipid content among prey fish specimens (Vuorinen et al. 2002; Keinänen et al. 2012). The average thiamine concentration of sprat and herring do not differ (Keinänen et al. 2012), but in autumn, when the lipid content of fish is at its highest, the thiamine concentration in more fatty sprat is lower than in herring (Vuorinen et al. 2002). Among both species, the thiamine concentration is lowest in the youngest specimens with the highest lipid content (Vuorinen et al. 2002; Keinänen et al. 2012, 2017).

Feeding on lipid-rich fish increases the lipid content and oxidative stress in salmonid tissues (Alvarez et al. 1998; Hemre and Sandnes 1999; Gélineau et al. 2001; Todorčević et al. 2009; Østbye et al. 2011). Thiamine deficiency results from an unbalanced diet containing an abundance of fatty prey fish, from which the supply of thiamine is insufficient in proportion to the supply of energy and unsaturated FAs (Keinänen et al. 2012, 2017). As a result of peroxidation and free radical oxidation of FAs (Tacon 1996; Lukienko et al. 2000; Spector 2000; Gibson and Zhang 2002), thiamine resources acquired during the feeding migration are depleted from salmon tissues during the long pre-spawning fasting period (Karlsson et al. 1999b; Koski et al. 2001).

**Fig. 2:**
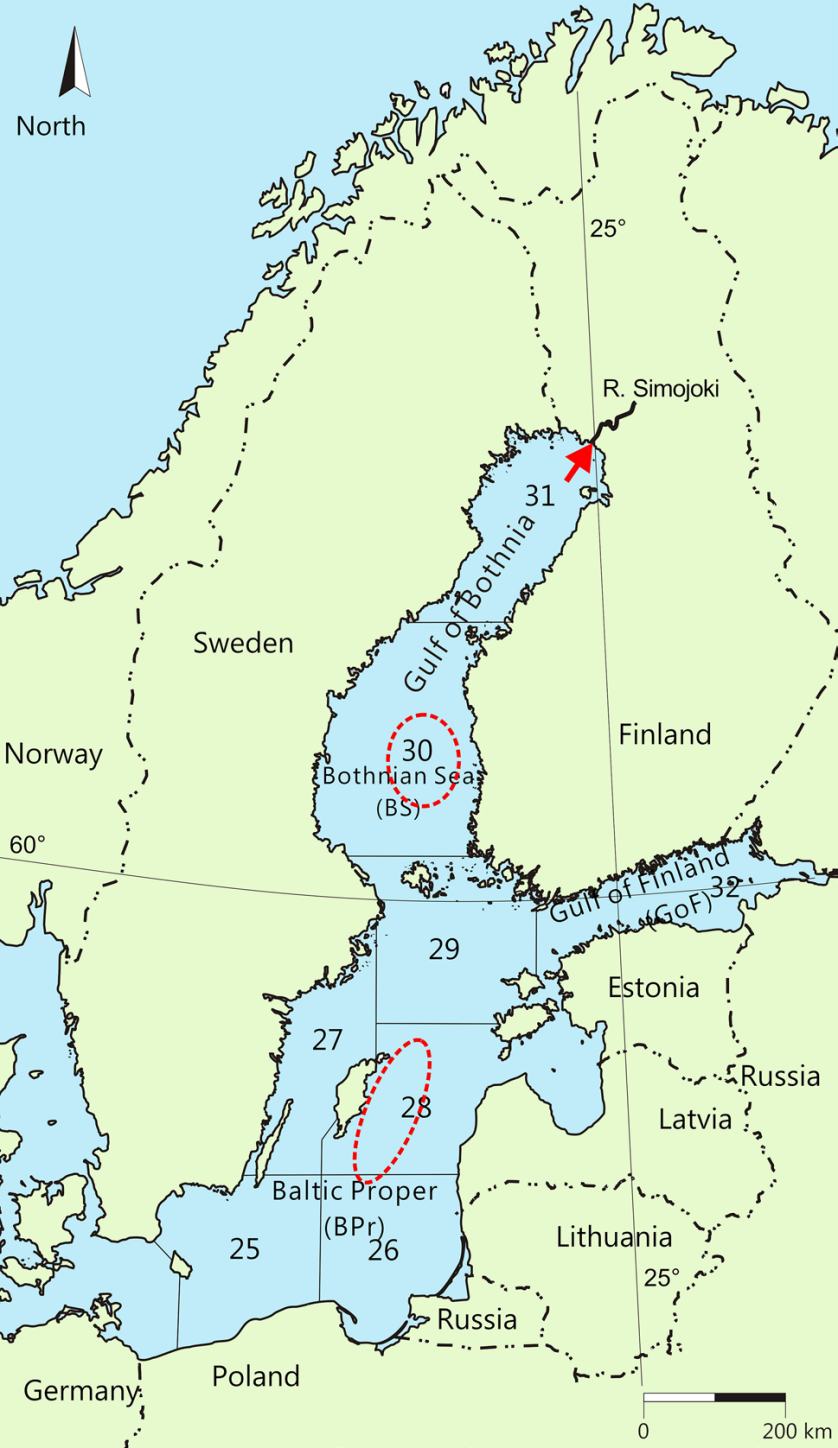
The Baltic Sea [with the subdivisions (SD 25–32) of the International Council for the Exploration of the Sea (ICES, origin of the map)]. Salmon of the study undergoing the feeding migration were sampled (ellipses with a broken line) from the Baltic Proper (BPr) from SD 28 and the Bothnian Sea (BS), SD 30. Spawning salmon females of the study had ascended the River Simojoki (arrow), one of the rivers in which salmon still reproduce naturally. The latitude of 60° and longitude of 25° are indicated

Most salmon in the Baltic Sea originate from rivers flowing into the northern areas of the sea, specifically from the rivers of the Gulf of Bothnia (ICES 2018a) (Fig. 2). All of these salmon stocks have suffered from M74 (Karlström 1999; Romakkaniemi et al. 2003; Keinänen et al. 2008; ICES 2018a). In the main feeding areas of salmon, over 90% of the salmon diet, both on a specimen number and a weight basis, has consisted of sprat and herring (Hansson et al. 2001). However, the relative abundances of these clupeids vary between feeding areas (Mikkonen et al. 2011). In the southern part of the Baltic Sea, the Baltic Proper (BPr), where the main spawning areas of sprat are located (Aro 1989), sprat has been the main prey of salmon (Hansson et al. 2001; Mikkonen et al. 2011; ICES 2018b). Herring is the dominant prey species in the northern feeding area, the Bothnian Sea (BS) (Hansson et al. 2001; Salminen et al. 2001; Mikkonen et al. 2011), where sprat do not reproduce due to the low water salinity (Aro 1989). Most salmon from the rivers of the northern Gulf of Bothnia, such as the River Simojoki (Fig. 2), extend their feeding migration to the BPr, but some appear to halt to feed in the BS (Aro 1989; Salminen et al. 1994).

The FA composition of Baltic sprat and herring has been compared by Røjbek et al. (2014) in the southwestern BPr and by Keinänen et al. (2017) in different and more northern areas of the Baltic Sea. In both of these investigations, analyses were performed on the whole body of clupeids, describing their actual FA composition as food for predatory fish, but only in the study by Keinänen et al. (2017), the analysed herring were of the smaller size that are preferred by salmon (Hansson et al. 2001; Mikkonen et al. 2011; Vuorinen et al. 2014a). Despite some intra-species variation between the sea areas and seasons, the FA composition of sprat and herring differed. The proportion of oleic acid (18:1n-9), the dominant monounsaturated fatty acid (MUFA), differed between the species, being higher in sprat, but did not differ between areas or seasons (Røjbek et al. 2014; Keinänen et al. 2017). Although the proportion of MUFAs in total was higher in sprat, the proportions of two minor MUFAs, palmitoleic acid (16:1n-7) and vaccenic acid (18:1n-7), were higher in herring (see Table 1 in Keinänen et al. 2017). The proportion of a polyunsaturated FA of the n-6 family (n-6 PUFAs), arachidonic acid (ARA, 20:4n-6), was higher in herring than in sprat, but did not differ between the areas (Keinänen et al. 2017). The proportion of n-6 PUFAs in total, apart from being higher in herring, was also higher in the BS. Hence, 18:1n-9 and ARA and their ratios could most probably serve as tracers indicating the principal dietary species (sprat vs. herring) and, thus, also the feeding area (BPr vs. BS) of Baltic salmon. The proportion of a saturated FA (SFA), myristic acid (14:0), could specifically serve as a tracer for the feeding area, as it has occurred in higher proportions in prey fish in the BPr than in the BS (Keinänen et al. 2017; Lind et al. 2018).

In terms of concentrations (lipid-weighted basis), due to their higher lipid content, sprat contained not only more of 18:1n-9 but also almost all FAs than herring. An exception was those minor FAs (all individual n-6 FAs and 16:1n-7 and 18:1n-7) that were characteristic of herring (Keinänen et al. 2017). Although 18:1n-9 was the most common FA in sprat, the concentration of the prevalent polyunsaturated FA (PUFA), docosahexaenoic acid (DHA, 22:6n-3), increased most clearly with an increase in the lipid content of prey fish (Keinänen et al. 2017). The DHA concentration and lipid content were highest in the youngest sprat (Keinänen et al. 2017) among prey fish specimens preferred by salmon (Mikkonen et al. 2011). Although 18:1n-9 also increases the vulnerability of fish to oxidative stress through free radical oxidation (Lukienko et al. 2000), a large part of the oxidative stress may originate from DHA, being a highly unsaturated FA (n-3 HUFA) with the highest number of double bonds and, thus, extremely prone to lipid peroxidation (Tacon 1996; Spector 2000).

The incidence of M74 has fluctuated from non-existent to extremely detrimental over the course of years and decades (Fig. 1) (Keinänen et al. 2014; ICES 2018a). M74 mortality has been high when the new year classes of sprat and sprat stock have been strong, and the biomass of salmon prey fish in the BPr has consequently been high (Karlsson et al. 1999a; Mikkonen et al. 2011). Apart from varying annually, the severity of thiamine deficiency, i.e., the thiamine concentration in the eggs of salmon, varies to some degree between rivers (Keinänen et al. 2008; ICES 2018a). However, it also can vary between female individuals of the same river within years, leading to mortality differences from insignificant to 100% between offspring groups (Amcoff et al. 1999; Lundström et al. 1999; Keinänen et al. 2000). These variations are thought to depend on the feeding area and consequently on feeding to a different extent on sprat and herring. A relationship between FAs and thiamine in the food web is probably the cause of M74, but this needs to be investigated, as stable isotope analysis (SIA) could not provide that information (Torniainen et al. 2017). Knowledge of the FA composition of salmon caught during their feeding migration that have fed in different areas, and thus on different diets, is needed to resolve the nutritional origin of this reproductive disorder of salmon. Fatty acid signature analysis has been used to assess the diet of Atlantic salmon (Budge et al. 2012; Skilbrei et al. 2015). Thus, the feeding area and the principal prey fish of salmon spawners could most probably be resolved by comparing the FA profiles of brood salmon with those of salmon caught from the different feeding areas.

The aim of the present study was to investigate whether the dietary habits of spawning salmon that have been feeding in the Baltic Sea could be resolved by FA signature analysis to relate the occurrence of the M74 syndrome to the principal prey species. This was accomplished, firstly, by examining (1) how sprat and herring differ in fatty acid composition, and (2) how the fatty acid profiles of salmon from the BPr and BS differ and whether their FA profiles reflect their main prey species. Secondly, differences were sought in the FA profiles (3) between spawning females from the year when M74 was prevalent and when M74 did not occur and (4) between females producing M74 yolk-sac fry and those that did not within a year when M74 did occur. The hypothesis was that the FA composition of spawning salmon with a low thiamine concentration in the eggs more closely resembles that of sprat and salmon caught from the BPr than that of herring and salmon caught from the BS. Thus, the study sought to provide evidence to support earlier findings (Mikkonen et al. 2011; Keinänen et al. 2012, 2017) that the principal dietary origin of the thiamine deficiency syndrome M74 in years with a high and moderate incidence has been abundant fatty sprat, which has thus far been concluded from statistics and different types of data analysis.

## Materials and methods

### Sprat and herring data

The FA data on sprat [*Sprattus sprattus* (L.)] and herring (*Clupea harengus membras* L.) from the northern BPr and from the BS, caught from autumn 2003 to spring 2004, were obtained from Keinänen et al. (2017). Whole fish were analysed to simulate the actual diet of salmon, as in earlier studies by Vuorinen et al. (2002, 2012) and Keinänen et al. (2012). The fish were pooled according to their age group and homogenised. Since adult salmon prefer prey fish of 4–15 cm in length and only rarely prey on fish > 20 cm (Hansson et al. 2001; Vuorinen et al. 2014a), all age groups of sprat but only the youngest age groups of herring (< 6 years) were considered as common prey of salmon (Mikkonen et al. 2011). Thus, the FA results for 6 homogenised pools of sprat, each containing 12–31 specimens, and 8 homogenised pools of 11–93 herring specimens were used for the present study.

### Sampling of salmon from the Baltic Sea

Baltic salmon (*Salmo salar* L.) were captured from the BPr in the Gotland Deep (hereafter BPr salmon) and the BS (hereafter BS salmon) (Fig. 2) during their feeding migration in October–December, in the same year (2004) as the prey fish samples in Keinänen et al. (2017). Immediately after removal from the net, salmon were killed by a blow on the head, their total length and weight were measured, scales were removed for age determination, they were opened (the liver was sampled for other analyses), and the whole salmon was sealed in a polyethylene bag and frozen (− 20 °C for 2 weeks). Fulton’s condition factor was calculated as: CF = 100 × *w* × *l*^−3^, where *w* = body weight (g) and *l* = total length (cm).

After partial thawing in the laboratory, a 20-g piece of epaxial white muscle was excised and immediately refrozen (− 80 °C) for FA analyses. Only 2nd sea-year salmon (9 from the BPr and 5 from the BS) were included in the present study. Both salmon males and females were included in the analyses, because the sex of non-reproducing salmon on the feeding migration caught in the sea in late autumn was not expected to affect the results.

### Sampling of salmon spawners and data on egg thiamine and M74 status

Following the outbreak of M74 at the beginning of the 1990s, the intensity of M74 has annually been monitored for salmon returning to spawn in the River Simojoki by measuring the thiamine concentrations in newly stripped unfertilized eggs and recording the M74-related female-specific yolk-sac fry mortalities (hereafter YSFM), as well as the proportion of those females that produce offspring suffering from M74 (hereafter M74 females vs. non-M74 females) (Fig. 1) (Keinänen et al. 2000, 2008, 2014; ICES 2018a). From this Finnish M74 monitoring programme of the Natural Resources Institute Finland (Luke, formerly the Finnish Game and Fisheries Research Institute) (Vuorinen et al. 2014b), 2nd sea-year salmon females of the River Simojoki from the reproductive period 1998/1999 (M74 year 1998; *N* = 21, of which 11 were M74 females) and from 2004/2005 (non-M74 year 2004; *N* = 6) were taken for FA analysis of dorsal muscle. Yolk-sac fry mortality and the concentration of unphosphorylated or free thiamine (hereafter THIAM) in unfertilized eggs, which has been used as a biomarker of M74 by Luke, were available from the M74 monitoring programme.

The salmon had been caught by trap-netting in June–July while ascending the River Simojoki to spawn. The salmon were marked with Carlin tags, and scales for age determination were taken. The fish were held in fibre-glass basins with through-flowing river water at the Keminmaa Hatchery of Luke until the stripping of eggs during the spawning period in October. For thiamine analysis, the eggs were stripped on a sieve to filter off the ovarian fluid from the sample. A sample of approximately 100 ml of eggs was taken and frozen as a sheet in a 500-ml ziplock bag with an identification code for the female salmon. The samples were immediately frozen (− 20 °C) and sent as airfreight to the laboratory packed in a cooler box with dry ice.

The total body weight before stripping of eggs and the total length of females were recorded, and the Fulton’s CF was calculated similarly to feeding salmon. An approximately 200-g piece of the left epaxial white muscle was dissected from below the dorsal fin, sealed in a polyethylene bag and immediately frozen. The muscle samples were sent frozen to the laboratory, where a 20-g subsample of each muscle sample was taken for FA analysis. The samples were stored for 4–6 weeks at − 80 °C until analysed.

Thiamine was analysed in the eggs of salmon according to Vuorinen et al. (2002) by high-performance liquid chromatography (HPLC). A laboratory control sample (a subsample of large salmon egg homogenate) was processed and analysed along with the samples for quality assurance. The HPLC analysis separated thiamine pyrophosphate (TPP), thiamine monophosphate (TMP) and THIAM, which were summed as total thiamine (TotTHIAM). Salmon sampled in 1998 were classified on the basis of the THIAM concentration in the eggs into M74 females (THIAM ≤ 0.65 nmol g^−1^) and non-M74 females. The symptoms caused by thiamine deficiency, as well as YSFM among offspring of the M74 females, had been observed and recorded.

### Fatty acids

For the FA analysis, the lipids of salmon muscle were extracted using the Schmid–Bondzynski–Ratzlaff method (ISO 1735/IDF 5: 2004). The FAs were analysed as in Keinänen et al. (2017) for sprat and herring. The extracted total lipids were transesterified in methanolic boron trifluoride according to Slover and Lanza (1979). The formed FA methyl esters (FAME) were analysed by gas–liquid chromatography (Agilent HP 5890) employing an HP Innowax capillary column (30 m, 0.32 mm × 0.5 µm) and flame-ionisation detection. Individual FAs were identified on the basis of their relative retention times. Fatty acids were analysed in the laboratory of the Finnish Food Safety Authority Evira. The FA results are presented as proportions (percentage values) based on their FAME peak area as a proportion of the total area of all the integrated chromatographic peaks. The FAs were grouped into SFAs, MUFAs and PUFAs, and the latter were further divided into the sums of n-3 PUFAs and n-6 PUFAs, similarly as in Keinänen et al. (2017). Among n-3 HUFAs, those n-3 PUFAs, DHA, eicosapentaenoic acid (EPA, 20:5n-3) and docosapentaenoic acid (DPA, 22:5n-3) were included that possess 20 carbon atoms and three double bonds at minimum (Tocher 2003). For the calculations, the 15 FAs were included for which the mean proportions were > 0.5%, in addition to DPA, which appeared to enrich in salmon, although its proportion in sprat and herring was < 0.5%.

### Statistical analyses

One-way ANOVA was applied to test the differences in the proportions of individual FAs, FA sums and some of their ratios in muscle between BPr and BS salmon, between female salmon spawners from the River Simojoki in the M74 year (1998) and the non-M74 year (2004), between M74 females and non-M74 females in the M74 year (1998) and, moreover, in the body characteristics (total length, weight and CF) and M74-related characteristics (the concentrations of thiamine components in eggs and YSFM) between the same study groups. One-way ANOVA (*P* < 0.05) was also used to compare the FA proportions between the feeding salmon and spawning salmon (2004) and to compare the proportional differences of FAs within groups. On comparing the FA differences between the feeding salmon from the BPr and BS and spawning salmon from the M74 year and non-M74 year, one-way ANOVA with Bonferroni adjustment for the group comparisons was applied. Pearson correlation coefficients were calculated between the concentration of all thiamine components and TotTHIAM of eggs, as well as YSFM and the proportions of muscle FAs, their sums and some of their ratios, and the size information for female salmon from the River Simojoki in the M74 year and the years 1998 and 2004 combined. For statistical calculations, the ratios of FAs typical for sprat or BPr salmon to FAs typical for herring or BS salmon were included. For correlation analysis, some ratios were included that have been reported in the literature in connection with thiamine deficiency in salmonids. In all these calculations, untransformed percentage values were used (Warton and Hui 2011).

Principal component analysis (PCA; Kvalheim and Karstang 1987) was used for the multivariate statistical comparisons of the FA profiles of salmon caught during their feeding migration from the BPr and BS with their main prey species, sprat and herring, and for the comparisons of the FA composition of sprat, herring, BPr salmon and BS salmon. Principal component analysis was also applied for the separation of salmon female spawners between the M74 year and the non-M74 year and within the M74 year on the basis of their FA profiles. Prior to the PCA, the FA data were arcsine transformed and subsequently standardised (variable deviations homogenised) to prevent the abundant components with large variance from dominating the analysis. Biplot graphs based on PCA were constructed to illustrate the compositional similarities/differences among and between the groups and to indicate correlations between the variables. Soft independent modelling of class analogy (SIMCA) was used to quantify the compositional differences between the fish groups at the *P *< 0.05 level (Wold and Sjöström 1977).

Principal component analysis and SIMCA were performed with Sirius software (ver. 8.5, Pattern Recognition Systems, Norway), and the other statistical analyses with Statistical Analysis System (ver. 9.4) software (SAS Institute Inc. 2008).

## Results

### Fatty acid composition of feeding salmon

The dominant FA in the muscle of salmon in both areas was 18:1n-9, and its proportion tended to be higher in BPr salmon than in BS salmon (Table 1). On the contrary, the proportion of 18:1n-7, the isomer of 18:1n-9, was significantly higher in BS salmon. The proportion of the second most prevalent FA, palmitic acid (16:0) tended to be higher in BPr salmon. In addition, the proportions of the next most common SFA, 14:0, and SFAs altogether were significantly higher in BPr than in BS specimens. The third most common FA in salmon muscle was DHA. Contrary to the most prevalent MUFA and SFA, the proportion of DHA, and consequently that of n-3 PUFAs, was higher in salmon from the BS, as was the total proportion of PUFAs (Table 1).

**Table 1:** Mean (± SE) proportions of FAs, their sums and some of their ratios in the muscle of 2nd sea-year salmon from the Baltic Proper (BPr, *N* = 9) and Bothnian Sea (BS, *N* = 5), and their total body length, weight and condition factor (CF)

	Baltic proper (BPr)	Bothnian Sea (BS)	*P*	*F*(1,12)
Saturated FAs (SFA)				
14:0	**3.62** ± **0.07**	3.17 ± 0.10	0.002	15.8
16:0	**18.45** ± **0.29**	17.60 ± 0.27	0.078	3.73
17:0	0.51 ± 0.02	0.52 ± 0.01	0.777	0.08
18:0	3.45 ± 0.05	3.38 ± 0.10	0.488	0.51
SFA	**26.85** ± **0.41**	25.14 ± 0.39	0.017	7.61
Monounsaturated FAs (MUFA)				
16:1n-7	4.30 ± 0.08	4.41 ± 0.09	0.405	0.74
17:1n-8	**0.62** ± **0.01**	0.41 ± 0.02	< 0.0001	128
18:1n-9	**21.70** ± **0.68**	19.67 ± 0.35	0.056	4.49
18:1n-7	3.01 ± 0.07	**4.64** ± **0.25**	< 0.0001	67.1
20:1n-9	1.07 ± 0.15	1.34 ± 0.07	0.227	1.62
MUFA	31.14 ± 0.84	31.14 ± 0.34	0.997	0.00
Polyunsaturated FAs (PUFA)				
18:2n-6	3.31 ± 0.06	**4.54** ± **0.06**	< 0.0001	180
18:3n-3	2.64 ± 0.06	2.56 ± 0.05	0.344	0.97
20:2n-6	0.74 ± 0.02	**1.28** ± **0.04**	< 0.0001	190
20:4n-6 (ARA)	0.60 ± 0.01	**0.74** ± **0.02**	< 0.0001	64.8
20:5n-3 (EPA)	5.80 ± 0.16	5.92 ± 0.11	0.615	0.27
22:5n-3 (DPA)	2.85 ± 0.09	**3.29** ± **0.13**	0.014	8.36
22:6n-3 (DHA)	14.76 ± 0.57	**17.05** ± **0.46**	0.019	7.33
PUFA	31.74 ± 0.74	**36.01** ± **0.61**	0.0002	26.3
n-3 PUFA	26.19 ± 0.75	**28.82** ± **0.52**	0.009	9.72
n-6 PUFA	5.55 ± 0.04	**5.97** ± **0.13**	< 0.0001	194
FA ratios				
14:0/ARA	**6.06** ± **0.17**	4.29 ± 0.21	< 0.0001	40.9
18:1n-9/ARA	**36.31** ± **1.31**	26.59 ± 0.98	0.000	25.5
Salmon body characteristics				
Total length (cm)	71.0 ± 1.5	70.9 ± 1.3	0.936	0.01
Body weight (kg)	3.64 ± 0.24	3.72 ± 0.31	0.844	0.04
CF	1.00 ± 0.02	1.04 ± 0.03	0.396	0.77

A significant difference (*P* value) and the *F* value (with the degrees of freedom in parentheses) in one-way ANOVA between the salmon groups is indicated by the larger mean being in bold face, when *P* < 0.1

The most conspicuous difference between the salmon of the two areas was that the proportions of all individual n-6 PUFAs, linoleic acid (18:2n-6), eicosadienoic acid (20:2n-6) and ARA, as well as n-6 PUFAs in total were clearly higher in BS than in BPr salmon (Table 1). Thus, the ratios of 18:1n-9 and 14:0 to ARA were clearly higher in BPr salmon.

Neither the size nor CF of the salmon significantly differed between the areas (Table 1).

### Reflection of the FA profile of prey fish in feeding salmon

The principal differences in FA composition between sprat and herring remained similar to those reported in Keinänen et al. (2017), although in the present study, only FA results for salmon prey fish from the BPr and BS were included, and prey fish from the Gulf of Finland were omitted. Sprat from the BPr and herring from the BS were clearly discriminated on the basis of their FA profiles when their FA proportions in spring and autumn were combined (additional Fig. 1). The FA profiles did not differ within the species between the areas, despite a slight shift (not shown).

**Fig. 3:**
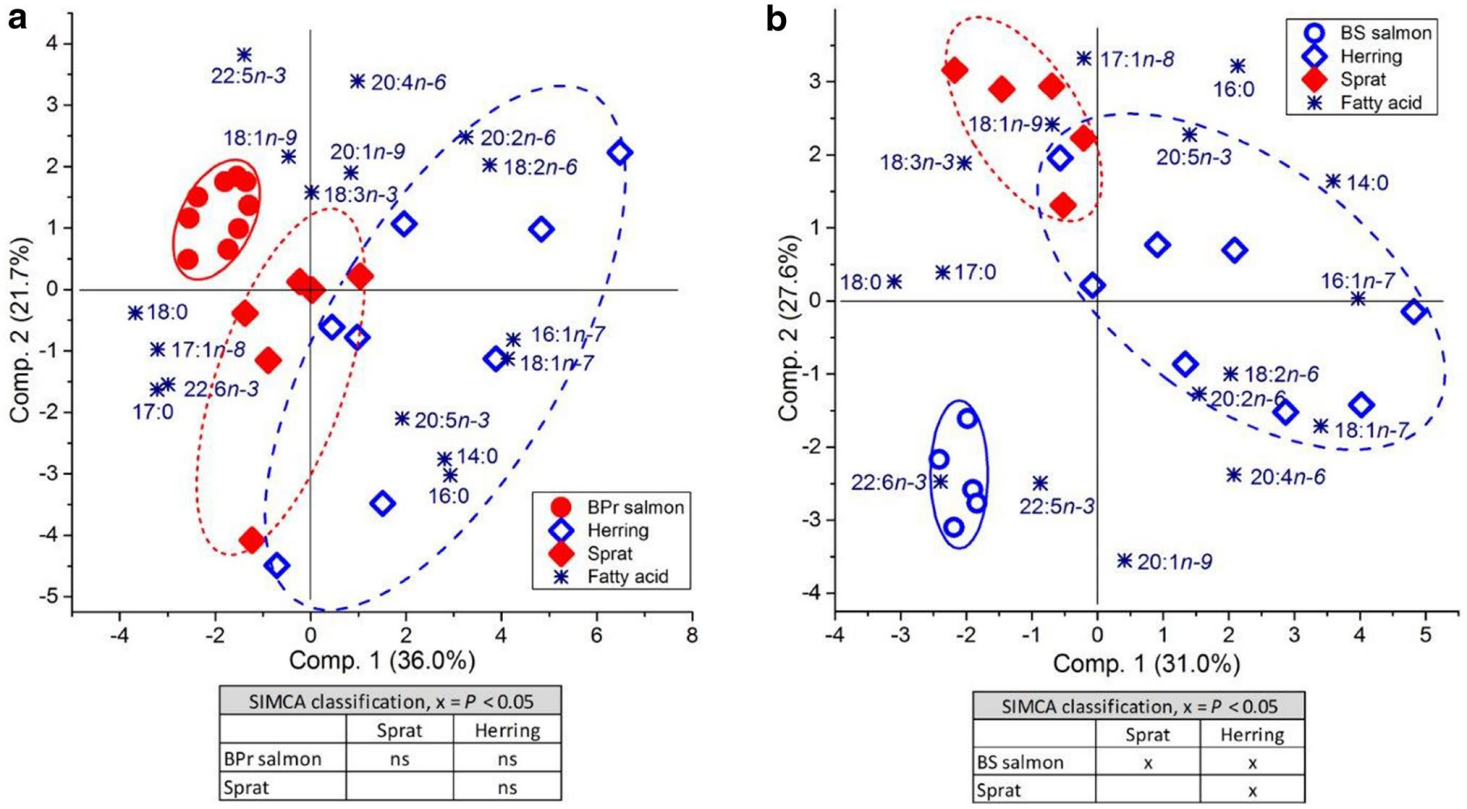
Biplots based on PCA and subsequent paired SIMCA test results for the proportions of FAs in a) 2nd sea-year salmon, sprat and herring from the Baltic Proper (BPr) and b) 2nd sea-year salmon, sprat and herring from the Bothnian Sea (BS). FAs were analysed from the muscle of salmon and whole body of sprat and herring. The closer the groups are located on the biplot, the more similar they are in terms of their FA composition. In the SIMCA contingency table, significant differences between the FA signatures of the two compared groups are marked by “x”. FA proportions of sprat and herring are from Keinänen et al. (2017)

Although BPr salmon, sprat and herring were not separated from each other by principal components 1 and 2, according to the SIMCA classification, the FA compositions of BPr salmon and sprat were closer to each other than those of BPr salmon and herring in the PCA biplot (Fig. 3a). On the contrary, the FA composition of BS salmon was slightly closer to the FA composition of herring than that of sprat, although BS salmon were separated from both according to the SIMCA analysis, as was sprat from herring (Fig. 3b). In both areas, 18:1n-9 was associated with sprat, whereas the minor MUFAs 16:1n-7 and 18:1n-7 and individual n-6 PUFAs were linked to herring. Although 18:1n-9 was also associated with BPr salmon, it had no association with BS salmon. Arachidonic acid was associated with salmon to a larger degree than other n-6 PUFAs, and DHA and DPA were clearly more linked to salmon than to prey fish. In both areas, the intraspecific variation in the FA composition, as revealed by the PCA biplots (Fig. 3a, b), was largest for herring, and the salmon FA compositions were the most uniform.

**Fig. 4:**
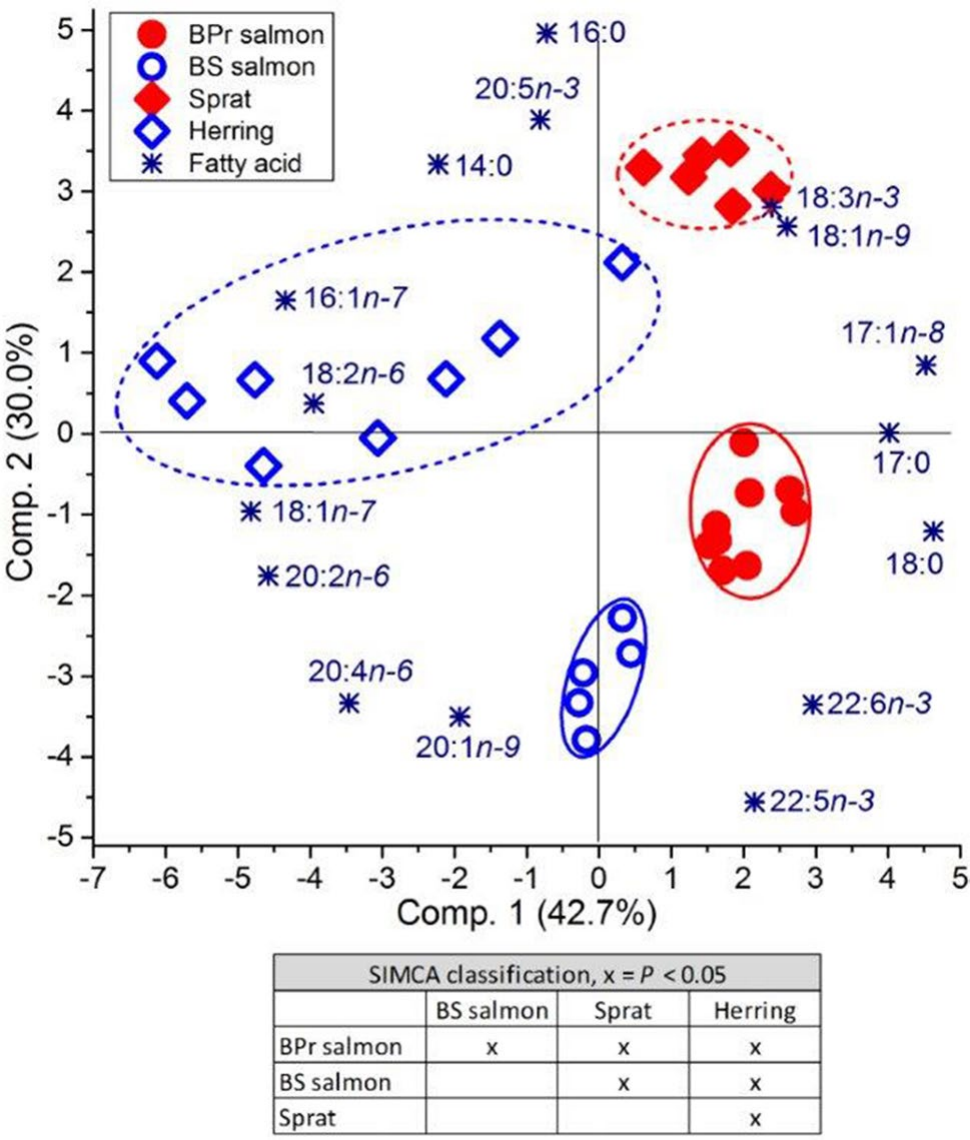
Biplot based on PCA and the results of the subsequent paired SIMCA test for the proportions of FAs in 2nd sea-year salmon from the Baltic Proper (BPr salmon) and the Bothnian Sea (BS salmon) and sprat and herring in autumn from the BPr and the BS

Sprat and herring were clearly separated from each other when combining the FA proportions of each species from both the BPr and the BS in autumn (Fig. 4). The FA profiles of BPr and BS salmon were likewise separated from each other and from those of sprat and herring (Fig. 4). However, BPr salmon and sprat, as well as BS salmon and herring, were associated with each other in the PCA biplot. Among all FAs, DHA was the one with the clearest association with salmon, both in the BPr and BS, and DPA had a clearer association with BS salmon than BPr salmon (Fig. 4). The prevalent MUFA 18:1n-9 and alpha-linolenic acid (18:3n-3) were linked to sprat, whereas individual n-6 PUFAs (18:2n-6, 20:2n-6 and ARA), as well as 18:1n-7 and 16:1n-7, were associated with herring. However, ARA had a clearer association with BS salmon than herring. Fatty acids 14:0, EPA and 16:0 were linked to prey fish. The overall intraspecific variation in the FA composition was larger for herring than for sprat (Fig. 4).

### Fatty acids of spawning salmon in relation to M74 and FA signatures of prey fish

#### Differences between the M74 year and non-M74 year

Among the most common FAs, the proportion of 16:0 in the muscle of spawning salmon in the M74 year (1998) and the non-M74 year (2004) was significantly lower (ANOVA, *F*(3,37) = 103.90, *P* < 0.0001) than in salmon feeding in either sea area, and SFAs in total were similarly lower. Conversely, the proportions of DHA and 18:1n-9 were higher overall in spawning salmon compared to feeding salmon (Tables 1, 2). When the muscle FA proportions of spawning salmon of the non-M74 year (2004) were compared with those of feeding salmon caught in 2004, the proportion of 16:0 was 25–29% lower and the proportion of SFAs in total 21–26% lower. On the contrary, the proportion of DHA was 26–46% higher and the proportions of 18:1n-9 and MUFAs were slightly higher in the muscle of spawning salmon in 2004 than that of feeding salmon in the same year.

**Table 2:** Mean (± SE) proportions of FAs, their sums and some of their ratios in the muscle of female 2nd sea-year salmon from the River Simojoki in the M74 year (1998, *N* = 21) and non-M74 year (2004, *N* = 6), and the total body length and weight as well as the condition factor (CF) and concentrations of different thiamine components (*TPP* thiamine pyrophosphate, *TMP* thiamine monophosphate, *THIAM* free thiamine) and total thiamine (TotTHIAM) in their eggs, and offspring mortality during the yolk-sac phase (female-specific yolk-sac fry mortality = YSFM)

Variable	M74 year (1998)	Non-M74 year (2004)	*P*	*F*(1,25)
Saturated FAs (SFA)				
14:0	2.61 ± 0.07	2.40 ± 0.23	0.248	1.40
16:0	12.97 ± 0.18	13.19 ± 0.53	0.614	0.26
17:0	0.57 ± 0.06	0.43 ± 0.02	0.204	1.71
18:0	3.48 ± 0.08	3.50 ± 0.15	0.928	0.01
SFA	20.12 ± 0.22	19.89 ± 0.68	0.672	0.18
Monounsaturated FAs (MUFA)				
16:1n-7	**4.43** ± **0.10**	3.80 ± 0.17	0.005	9.65
17:1n-8	0.69 ± 0.06	0.48 ± 0.04	0.100	2.91
18:1n-9	**24.31** ± **0.51**	22.21 ± 0.67	0.050	4.26
18:1n-7	3.34 ± 0.19	3.55 ± 0.23	0.594	0.29
20:1n-9	1.49 ± 0.06	1.43 ± 0.52	0.849	0.04
MUFA	**34.81** ± **0.44**	32.14 ± 0.80	0.009	8.16
Polyunsaturated FAs (PUFA)				
18:2n-6	3.75 ± 0.12	3.40 ± 0.07	0.142	2.30
18:3n-3	2.24 ± 0.03	2.34 ± 0.08	0.126	2.51
20:2n-6	0.73 ± 0.05	0.77 ± 0.03	0.614	0.26
20:4n-6 (ARA)	0.63 ± 0.03	**0.84** ± **0.05**	0.001	13.5
20:5n-3 (EPA)	4.72 ± 0.11	**6.04** ± **0.21**	< 0.0001	32.3
22:5n-3 (DPA)	0.29 ± 0.02	**4.54** ± **0.30**	< 0.0001	775
22:6n-3 (DHA)	18.79 ± 0.48	**21.50** ± **1.30**	0.024	5.81
PUFA	32.03 ± 0.48	**40.36** ± **1.66**	< 0.0001	45.0
n-3 PUFA	26.03 ± 0.56	**34.41** ± **1.59**	< 0.0001	39.3
n-6 PUFA	5.10 ± 0.17	5.01 ± 0.12	0.782	0.08
FA ratios				
14:0/ARA	**4.27** ± **0.21**	2.99 ± 0.43	0.009	8.01
18:1n-9/ARA	**39.46** ± **1.50**	27.16 ± 2.06	0.0004	16.6
Salmon characteristics				
TPP (nmol g^−1^)	0.410 ± 0.030	0.347 ± 0.033	0.299	1.13
TMP (nmol g^−1^)	0.142 ± 0.011	**0.234** ± **0.023**	0.001	15.2
THIAM (nmol g^−1^)	0.942 ± 0.173	**1.733** ± **0.363**	0.046	4.41
TotTHIAM (nmol g^−1^)	1.494 ± 0.192	**2.314** ± **0.396**	0.060	3.87
YSFM (%)	47 ± 9	18 ± 16	0.160	2.10
Length (cm)	83.9 ± 1.3	88.2 ± 1.8	0.137	2.36
Weight (g)	5.48 ± 0.27	5.92 ± 0.51	0.523	0.42
CF	0.91 ± 0.02	0.88 ± 0.02	0.408	0.71

A significant difference (*P* value) and the *F* value (with the degrees of freedom in parentheses) in one-way ANOVA between the salmon groups is indicated by the larger mean being in bold face, when *P* < 0.1

In the muscle of spawning salmon, the proportions of 18:1n-9 and 16:1n-7 were higher and the proportions of DHA, EPA and DPA lower in the M74 year than in the non-M74 year (Table 2). Consequently, MUFAs comprised the predominant FA group in salmon in the M74 year, and even more conspicuously, PUFAs overall, in addition to n-3 PUFAs, in the non-M74 year.

Among n-6 PUFAs, the only significant difference between the spawning years was in the proportion of ARA, which was lower in the M74 year than in the non-M74 year (Table 2). The ratio 18:1n-9/ARA was significantly higher in the M74 year, as was 14:0/ARA.

**Fig. 5:**
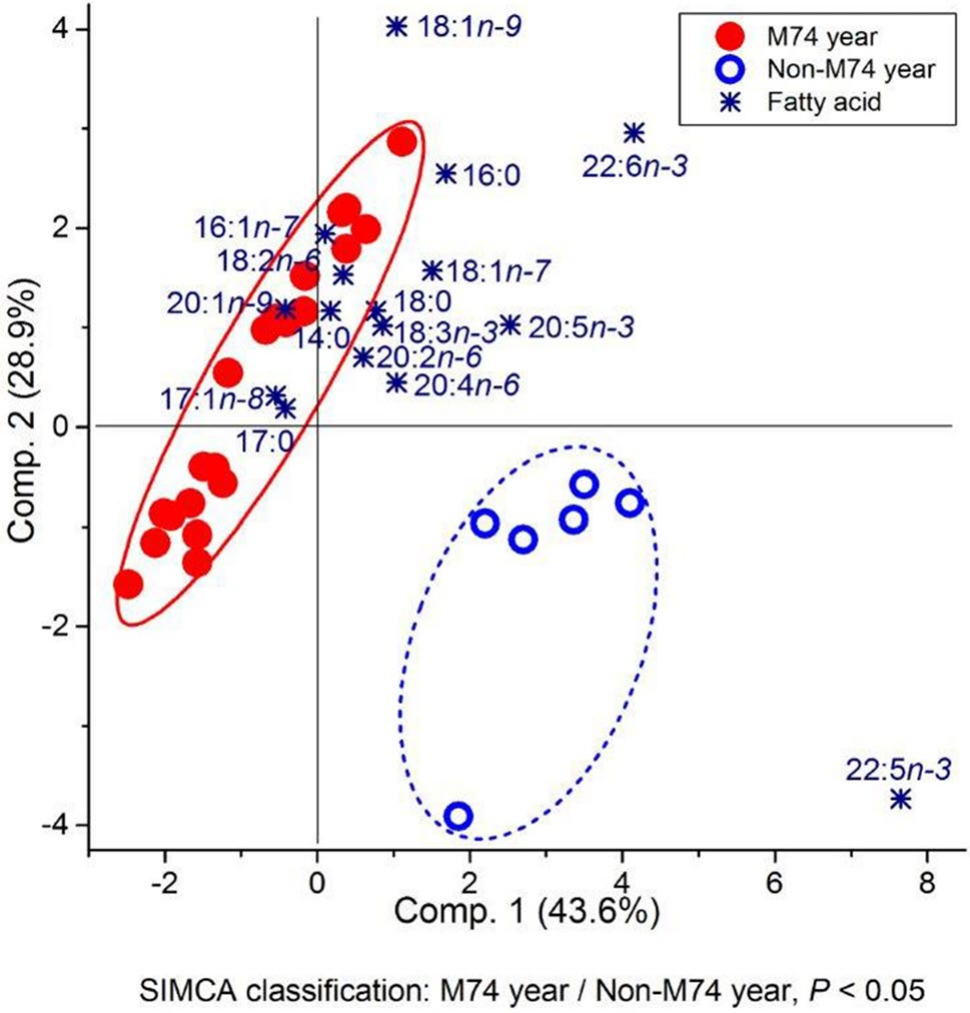
Biplot based on PCA and subsequent paired SIMCA test result for 2nd sea-year female salmon from the M74 year (1998) and non-M74 year (2004) classified on the basis of the proportions of muscle FAs

Principal component analysis and the associated SIMCA classification revealed that the FA composition of female salmon in the M74 year differed significantly from that in the non-M74 year (Fig. 5). Salmon of the M74 year were enriched in most of the MUFAs, including 18:1n-9, and also in the different SFAs, all being characteristic FAs for storage neutral lipid. Highly unsaturated FAs in general were associated with the females of the non-M74 year, and among these FAs, DPA had most power in separating the females of the non-M74 year from the females of the M74 year. Arachidonic acid and EPA were also clearly associated, and DHA weakly associated with the females of the non-M74 year.

The mean THIAM concentration of eggs in the non-M74 year was almost twice as high as in the M74 year. In addition to THIAM, the concentrations of TMP and TotTHIAM were higher in salmon eggs in the non-M74 year than in the M74 year, but that of TPP did not differ (Table 2). In both years, the concentration of THIAM was on average highest of the thiamine components. The concentration of TMP was lower than that of TPP.

#### Differences within the M74 year between M74 females and non-M74 females

Within the M74 year (1998), the clearest differences between M74 females and non-M74 females were in 18:1n-7, ARA and 14:0. The proportions of 18:1n-7 and ARA were significantly lower, and that of 14:0 was significantly higher in M74 females compared to non-M74 females (Table 3). Although the difference in the proportion of 18:1n-9 between the female groups was not significant, its tendency to be higher in M74 females affected its ratios to 20:2n-6, ARA and n-6 PUFAs by increasing these ratios in M74 females. The clearest differences among the ratios were in 18:1n-9/ARA and 14:0/ARA (Table 3).

**Table 3:** Mean (± SE) proportion of FAs, their sums and some of their ratios in the muscle of female 2nd sea-year salmon spawners from the River Simojoki in 1998 classified as M74 females (*N* = 11) and non-M74 females (*N* = 10), as well as the total body length, weight and condition factor (CF), and the concentrations of different thiamine components (*TPP* thiamine pyrophosphate, *TMP* thiamine monophosphate, *THIAM* free thiamine) and total thiamine (TotTHIAM) in their eggs, and female-specific offspring mortality during the yolk-sac phase (YSFM)

Variable	M74 females	Non-M74 females	*P*	*F*(1,19)
Saturated FAs (SFA)				
14:0	**2.77** ± **0.09**	2.42 ± 0.08	0.009	8.46
16:0	12.87 ± 0.30	13.08 ± 0.19	0.584	0.31
17:0	0.60 ± 0.08	0.54 ± 0.09	0.603	0.28
18:0	3.42 ± 0.11	3.56 ± 0.11	0.367	0.85
SFA	20.16 ± 0.35	20.07 ± 0.27	0.841	0.04
Monounsaturated FAs (MUFA)				
16:1n-7	4.37 ± 0.08	4.49 ± 0.19	0.574	0.33
17:1n-8	0.75 ± 0.09	0.63 ± 0.10	0.386	0.79
18:1n-9	24.94 ± 0.79	23.61 ± 0.58	0.193	1.82
18:1n-7	2.93 ± 0.31	**3.79** ± **0.11**	0.021	6.33
20:1n-9	1.43 ± 0.06	1.55 ± 0.09	0.300	1.13
MUFA	34.96 ± 0.56	34.64 ± 0.73	0.728	0.12
Polyunsaturated FAs (PUFA)				
18:2n-6	3.68 ± 0.11	3.82 ± 0.22	0.584	0.31
18:3n-3	2.22 ± 0.04	2.25 ± 0.03	0.562	0.35
20:2n-6	0.67 ± 0.02	0.79 ± 0.10	0.236	1.50
20:4n-6 (ARA)	0.58 ± 0.02	**0.69** ± **0.05**	0.026	5.86
20:5n-3 (EPA)	4.57 ± 0.17	4.88 ± 0.12	0.170	2.04
22:5n-3 (DPA)	0.27 ± 0.01	0.31 ± 0.03	0.280	1.24
22:6n-3 (DHA)	18.46 ± 0.58	19.15 ± 0.80	0.493	0.49
PUFA	31.38 ± 0.71	32.75 ± 0.60	0.162	2.12
n-3 PUFA	25.53 ± 0.75	26.58 ± 0.86	0.366	0.86
n-6 PUFA	4.93 ± 0.12	5.30 ± 0.34	0.303	1.12
FA ratios				
14:0/ARA	**4.85** ± **0.23**	3.63 ± 0.24	0.002	13.8
18:1n-9/ARA	**43.56** ± **1.68**	34.95 ± 1.64	0.002	13.4
Salmon characteristics				
TPP (nmol g^−1^)	0.367 ± 0.044	0.458 ± 0.037	0.136	2.42
TMP (nmol g^−1^)	0.113 ± 0.008	**0.173** ± **0.016**	0.003	11.4
THIAM (nmol g^−1^)	0.343 ± 0.045	**1.602** ± **0.212**	< 0.0001	36.8
TotTHIAM (nmol g^−1^)	0.824 ± 0.075	**2.232** ± **0.224**	< 0.0001	38.4
YSFM (%)	**87.1** ± **5.8**	3.6 ± 0.7	<0.0001	184
Length (cm)	**86.5** ± **1.5**	80.9 ± 1.8	0.026	5.88
Weight (g)	**6.01** ± **0.32**	4.90 ± 0.38	0.038	4.97
CF	0.92 ± 0.02	0.91 ± 0.02	0.689	0.16

A significant difference (*P* value) and the *F* value (with the degrees of freedom in parentheses) in one-way ANOVA between the salmon groups is indicated by the larger mean being in bold face, when *P* < 0.1. In the eggs of the M74 females, the THIAM concentration was ≤ 0.65 nmol g^−1^

**Fig. 6:**
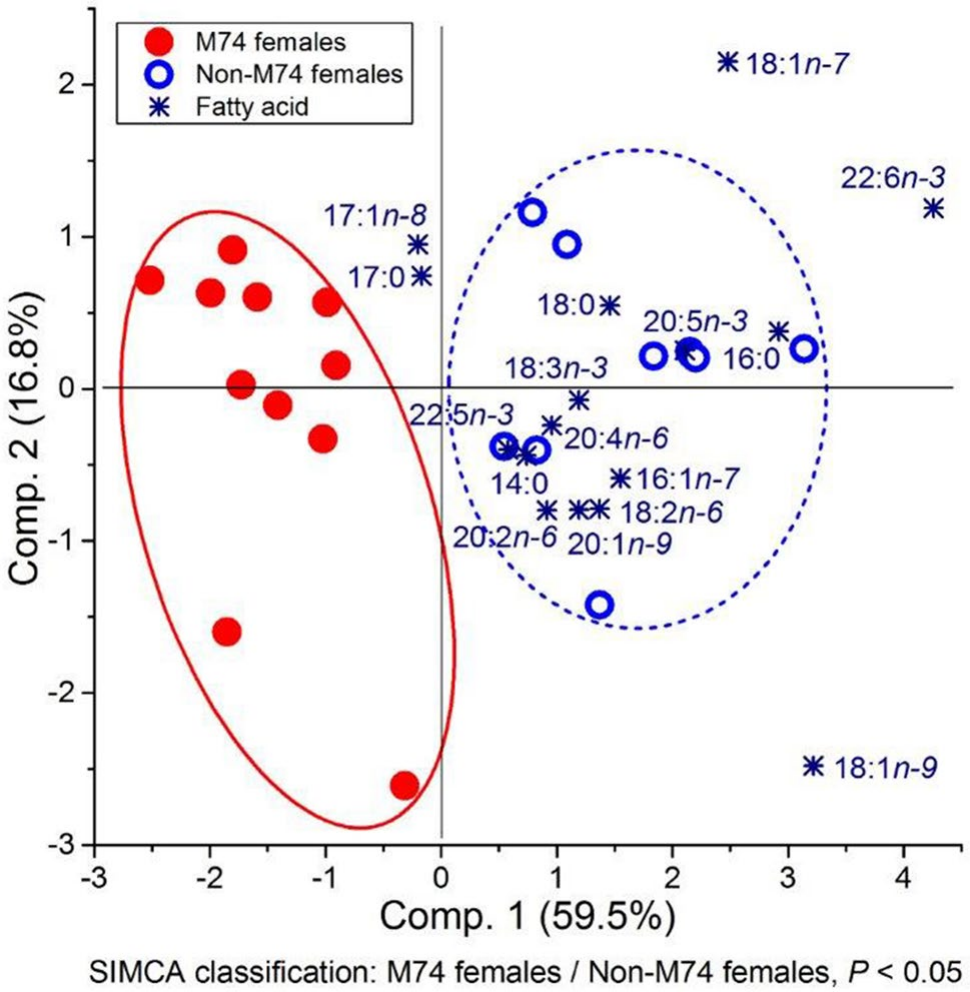
Biplot based on PCA and subsequent paired SIMCA test result for 2nd sea-year female salmon of M74 females and non-M74 females in the M74 year (1998) classified on the basis of the proportions of muscle FAs

The two groups, M74 females and non-M74 females, were separated from each other according to the SIMCA classification (Fig. 6). In the PCA biplot, DHA and 18:1n-7 were most clearly associated with non-M74 females, as also were most of the PUFAs.

The concentrations of egg THIAM and TotTHIAM, as well as YSFM, differed very clearly between the two female groups (Table 3). In non-M74 females, THIAM constituted the largest part of TotTHIAM. The concentration of TMP was also higher in non-M74 females, but that of TPP did not differ between the groups. M74 females were larger than non-M74 females.

#### Relationship of thiamine, mortality and body size with fatty acids

In the M74 year (1998), YSFM among the offspring of all female salmon had a highly significant negative correlation with the concentrations of THIAM (*r* = − 0.811, *P* < 0.0001) and TotTHIAM (*r* = − 0.805, *P* < 0.0001) in unfertilized eggs and also a correlation with the TMP (*r* = − 0.539, *P* = 0.012), but not with the TPP concentration (*r* = − 0.283, *P* = 0.214). The total length and body weight of females had a negative correlation with the THIAM concentrations and positive with YSFM (Table 4), indicating that M74 females were larger than non-M74 females.

**Table 4:** Pearson correlation coefficients (with the *P* value below) for female-specific offspring mortality during the yolk-sac phase (YSFM) and the free thiamine concentration (THIAM) in unfertilized eggs with the proportions of muscle FAs and some of their ratios in 2nd sea-year female salmon spawners (*N* = 21) from the River Simojoki in 1998, as well as with the total body weight and length

Variable	YSFM	*P*	THIAM	*P*
14:0	0.483	0.026	− 0.563	0.008
18:1n-7	− 0.379	0.090	0.418	0.059
20:4n-6 (ARA)	− 0.479	0.028	0.739	0.0001
n-6 PUFA	− 0.278	0.223	0.370	0.099
14:0/ARA	0.610	0.003	− 0.729	0.0002
18:1n-9/20:2n-6	0.443	0.044	− 0.493	0.023
18:1n-9/ARA	0.559	0.008	− 0.706	0.0003
18:1n-9/n-6 PUFA	0.387	0.083	− 0.479	0.028
DHA/EPA	0.125	0.589	− 0.444	0.044
Length	0.532	0.013	− 0.695	0.001
Weight	0.498	0.022	− 0.646	0.002

Only those FAs and ratios were included in the table for which either *P* value was < 0.1. All correlation results are presented in the Supplement Table

Among the FAs, the proportion of ARA had the strongest and most significant positive correlation with the THIAM concentration and negative correlation with YSFM (Table 4). The proportion of ARA had nearly as strong a correlation with the TotTHIAM concentration, but did not correlate with the concentrations of TPP or TMP (Supplement Table). Other FAs whose proportions showed positive correlations of some degree with the THIAM concentration were 18:1n-7 and n-6 PUFAs in total.

The proportion of 14:0 was positively correlated with YSFM and negatively correlated with the concentration of THIAM, TMP and TotTHIAM. The proportion of 18:1n-9 had also a negative correlation, but only with the phosphorylated thiamine components (Supplement Table).

The FA ratios representing the proportions of sprat and herring in the diet, 18:1n-9/ARA, 18:1n-9/20:2n-6 and 18:1n-9/n-6 PUFAs, or the feeding area, 14:0/ARA, had significant negative correlations with the THIAM (Table 4) and TotTHIAM and TMP concentrations (Supplement Table). Both 18:1n-9/ARA and 14:0/ARA had the strongest correlation with the concentration of THIAM from among the thiamine components (Table 4), whereas 18:1n-9/20:2n-6 and 18:1n-9/n-6 PUFAs had the strongest correlation with the TMP concentration (Supplement Table). A significant negative correlation with the THIAM concentration was also detected for DHA/EPA (Table 4).

When the M74 and the non-M74 year were both included in the correlation analysis (data not shown), YSFM among the FAs only correlated with the proportion of ARA (*r* = − 0.360, *P* = 0.065, *N *= 27). The proportion of ARA still had the strongest positive correlation with the THIAM (*r* = 0.554, *P* = 0.003, *N *= 27) and TotTHIAM (*r* = 0.518, *P* = 0.006, *N *= 27) concentrations. The FA ratios 18:1n-9/ARA and 14:0/ARA correlated strongly and negatively with the concentration of THIAM (*r* = − 0.568, *P* = 0.002, *N *= 27 and *r* = − 0.509, *P* = 0.007, *N *= 27, respectively) and positively with YSFM (*r* = 0.445, *P* = 0.020, *N *= 27 and *r* = 0.438, *P* = 0.022, *N *= 27, respectively).

## Discussion

### Connection of M74 with the diet and feeding area on the basis of the fatty acid signature

The FA profile of M74 females within the M74 year (1998) more closely resembled the FA profile of sprat and BPr salmon than that of herring and BS salmon. This provides evidence for thiamine deficiency being a result of preying abundantly on sprat in the southern Baltic Sea (Mikkonen et al. 2011; Keinänen et al. 2012). The significant differences between the two groups of spawners, M74 females and non-M74 females, in the M74 year remained in the herring-specific (or partly BS-specific) 18:1n-7 and ARA, and in 14:0, a signature of feeding in the BPr (Keinänen et al. 2017; Lind et al. 2018). Among the FA ratios tracing sprat (or BPr) (Røjbek et al. 2014; Keinänen et al. 2017) and herring (or BS), taking into account the ratios for which the differences were significant between BPr and BS salmon, and between salmon of the M74 year and the non-M74 year, the clearest differences were in the ratios 18:1n-9/ARA and 14:0/ARA. The FAs specific to herring better discriminated the salmon diet than 18:1n-9, probably because of some resemblance between the FA profiles of salmon and sprat. Salmon in both sea areas and sprat showed a high proportion of 18:1n-9, which is characteristic of storage lipids (Tocher 2003).

The results of the correlation analysis between the FA proportions and the egg THIAM concentration of females in the M74 year (1998) were consistent with the FA data discriminating M74 females from non-M74 females. The high proportion of ARA, best signaling a herring diet (Keinänen et al. 2017), was related to a higher THIAM concentration in eggs and lower YSFM among the offspring. On the contrary, a high ratio of 18:1n-9/ARA indicated that thiamine deficiency causing high YSFM is related to a higher proportion of dietary sprat. As the proportion of 14:0 was increased by feeding in the BPr (Keinänen et al. 2017; Lind et al. 2018), its positive correlation with YSFM and negative correlation with the concentration of THIAM provide evidence of M74 salmon with a higher proportion of 14:0 having been feeding in the BPr. According to Mikkonen et al. (2011), in the BPr, the principal prey for salmon spawners of the year 1998 had been sprat.

The egg THIAM concentration also correlated positively with the proportion of 18:1n-7, tracing herring as a species that eats benthic invertebrates (Keinänen et al. 2017; Happel et al. 2018). However, ARA among all FAs only correlated with the concentration of THIAM, the biomarker of M74, and not with the concentrations of TPP and TMP. The biologically active coenzyme form of thiamine is TPP, the concentration of which, consistently with the present study, has not varied with regard to YSFM (e.g. Vuorinen and Keinänen 1999). No biological role has been found for TMP, which is the intermediate molecule in thiamine metabolism (Lonsdale 2006). The correlation of ARA with the THIAM concentration was also strongest. Conversely, 18:1n-9 did not correlate at all with the THIAM concentration, but instead correlated negatively with the concentrations of the phosphorylated forms of thiamine. This may merely reflect a coincidental opposite change in the concentration (i.e. amount) of 18:1n-9 to that of body lipid and DHA, as was found in prey fish, particularly in sprat (Keinänen et al. 2017). A negative correlation of the ratio DHA/EPA with the THIAM concentration is consistent with earlier findings of FAs in thiamine-deficient salmonid eggs, in which higher DHA/EPA ratios has been reported in connection with both M74 (Pickova et al. 1998, 2003) and the respective thiamine deficiency of salmonids in the Great Lakes of North America, known as early mortality syndrome (EMS) (Czesny et al. 2009).

### The cause of the differences between the M74 year and non-M74 year

Spawning salmon clearly differed between the M74 year (1998) and the non-M74 year (2004) on the basis of their FA profiles, despite the incidence of M74 only being moderate in 1998. The overall average (± SE) YSFM of salmon from rivers of the north-eastern Gulf of Bothnia in the Finnish M74 monitoring (Keinänen et al. 2014; Vuorinen et al. 2014b) in the M74 year (56 ± 5%, *N *= 80) was over ten times higher than in the non-M74 year (5 ± 3%, *N* = 32), and the mean (± SE) THIAM concentration in unfertilized eggs (0.84 ± 0.11 nmol g^−1^, *N* = 76) was only a third of that in the non-M74 year (2.54 ± 0.25 nmol g^−1^, *N* = 32) (Keinänen et al. 2008; ICES 2018a). As not all spawners monitored for M74 were analysed for FAs, our results differ slightly, but with YSFM still being 2.6 times higher and the mean egg THIAM concentration on average 1.8 times lower in the M74 year than in the non-M74 year. Most probably, the actual inter-annual differences in the FA profiles of salmon have likewise been greater than were observed in the present study. Rather large variation in the THIAM concentration of eggs of females, particularly in the M74 year (range 0.14–3.31 nmol g^−1^), indicated differences in the feeding preferences or areas, although intra-specific differences in lipid metabolism are to some degree also possible (Sargent et al. 2002). However, still larger variation in the THIAM concentrations in M74 monitoring has been detected in years when moderate M74 mortalities have been recorded among offspring of salmon of the River Simojoki, e.g., in 2016/2017 from 0.10 up to 13 nmol g^−1^ (ICES 2018a).

The smaller differences in the muscle FA profile between M74 females and non-M74 females in 1998 than in females between the years 1998 and 2004 are consistent with fish stock changes in the Baltic Sea (Mikkonen et al. 2011). Those 2nd sea-year salmon that returned to spawn in 1998 had, during their two preceding feeding years in the sea, overall fed more on sprat, and especially abundantly on young sprat, than spawners of the year 2004. Young sprat were very numerous in the salmon diet during 1996–1998 (Hansson et al. 2001), and more numerous than in the two feeding migration years of 2004 spawners, i.e., in 2002–2004 (ICES 2018b). The large temporal variability in the abundance of sprat has depended on the stock strength of its principal predator, the cod (*Gadus morhua*) (Mikkonen et al. 2011), and the stock size of the cod increased before that latter time period (ICES 2018b).

### Effects of the pre-spawning period on the muscle fatty acid composition

The FA profile in the muscle of spawning salmon appeared to reflect their fasting during the spawning migration and the effects of exogenous vitellogenesis, besides reflecting the FA composition of their diet. While swimming from the feeding areas to the spawning grounds, salmon stop feeding long before spawning (Fleming and Einum 2011), and salmon of the northern Gulf of Bothnia rivers approximately 4 months before (Vuorinen et al. 2014a). Pre-spawning fasting was seen in the 2nd sea-year female salmon spawners of the River Simojoki as a loss of ca. 11% of the body weight between arrival at the home river and the spawning time (Mikkonen et al. 2011). In addition to energy reserves used for maintaining the basal metabolism and for swimming, body nutrients are incorporated into developing oocytes. However, particularly concerning HUFAs, the muscle and egg FA composition of spawning female salmon of the River Simojoki were similar (Torniainen et al. 2017).

Of the three dominant FAs, the proportion of 16:0 appeared to decrease most in the muscle during the pre-spawning fasting, and the proportion of SFAs altogether decreased nearly as much. According to Tocher (2003), 18:1n-9, in addition to 16:0, is heavily catabolized for energy in fish species such as salmonids, specifically during vitellogenesis. In the present study, the proportions of 18:1n-9 and MUFAs were slightly higher in spawning than in feeding salmon. This small proportional increase may be due to the prominent decrease in the percentage of 16:0 and thus SFAs as a whole, but in any case, 18:1n-9 and MUFAs had not been catabolized as much as SFAs. In steelhead trout (*Oncorhynchus mykiss*) migrating to spawn, SFAs and MUFAs were selectively depleted, but PUFAs were conserved (Penney and Moffitt 2015). Because the storage lipids accumulated during the feeding migration in muscle and viscera are depleted during fasting (Corraze and Kaushik 1999), the proportion of lipids in body structures, such as membranes, is emphasised. In coldwater fish, DHA and EPA are among the most important components of membranes (Corraze and Kaushik 1999; Sargent et al. 2002). Consistently, the proportion of DHA in muscle was 1.3–1.5 times higher in spawning, leaner salmon than in feeding salmon, apparently because it is not easily used as metabolic fuel (Tocher 2003). However, 18:1n-9 remained as the most common FA in spawning salmon in both the M74 year and the non-M74 year. Due to a large decrease in the proportion of 16:0, DHA was the second most common FA in spawning salmon, differently from feeding salmon.

### Reflection of the FA composition of prey in feeding salmon

The main FA differences of prey fish were manifested in the muscle of 2nd sea-year salmon caught during their feeding migration from the two sea areas, despite the sprat stock being smaller during the two preceding years than had ever been recorded since the beginning of the 1990s and the outbreak of the M74 syndrome (ICES 2018a). Although the diet of BPr salmon of the present study had evidently included less sprat and more herring than during the years with the highest M74 incidence (Mikkonen et al. 2011), dietary sprat was reflected as a higher proportion of 18:1n-9 in BPr salmon muscle compared to BS salmon. The BPr is a three times larger basin and a physically, chemically and biologically more variable area than the BS (HELCOM 2010; Mikkonen et al. 2011). Consequently, individual salmon returning to their spawning river may have fed in different feeding areas, resulting in the observed variability in their FA composition. As salmon apparently prey on the most available species of an appropriate size (Hansson et al. 2001; Vuorinen et al. 2014a), BPr salmon have mainly fed on sprat and less on herring, but in variable relative proportions depending on the subarea (Jacobson et al. 2018), whereas BS salmon have fed almost exclusively on herring (Mikkonen et al. 2011; Keinänen et al. 2012). In the BPr, the proportions of sprat and herring (Jacobson et al. 2018), and consequently the lipid content and energy density, in the salmon prey biomasses differed between the subareas during the observation period, i.e., since the 1970s (Keinänen et al. 2012). Individual salmon also possibly feed in more than one BPr subarea, although the food of the last months before their capture is supposedly reflected best in the FA composition (Skilbrei et al. 2015).

The FA profile of salmon was generally more similar to that of sprat than herring, 18:1n-9 being the dominant individual FA in both sprat and salmon (cf. Szlinder-Richert et al. 2010; Usydus et al. 2012; Røjbek et al. 2014; Keinänen et al. 2017). However, the similarity between salmon and herring was greater in the BS, where the dominant prey is herring (Hansson et al. 2001; Salminen et al. 2001; Vuorinen et al. 2014a). In BPr salmon, the second most prevalent FA was 16:0, whereas in BS salmon, the proportion of DHA was close to that of 16:0. The proportion of n-3 PUFAs, in addition to n-6 PUFAs, was higher in BS salmon than in BPr salmon, although Keinänen et al. (2017) reported that their proportions did not significantly differ between sprat and herring. As 18:2n-6 is characteristic of microalgae in freshwaters (Sargent et al. 2002), its higher proportion in BS herring and consequently in BS salmon may partly be associated with a lower environmental salinity than that prevailing in the BPr (HELCOM 2010). Contrary to the proportion of 18:2n-6, which is the metabolic precursor of ARA, the proportion of ARA did not differ in prey fish between the study areas (Keinänen et al. 2017). Moreover, ARA was according to PCA more closely linked to salmon than to herring, and in prey fish its proportion was higher in spring than after the intensive growth season in autumn. The differences between the proportions of 18:2n-6 and ARA apparently indicate ongoing metabolic processes, although ARA, among other long-chain PUFAs, has been considered as an essential FA (Parrish 2009), which can only to a limited degree be biosynthesized in salmon (Zheng et al. 2005; Morais et al. 2009).

The wider variability in the FA composition between individual herring than between specimens of solely zooplanktivorous sprat (Casini et al. 2004) apparently results from herring feeding on various food organisms, ranging from zooplankton to benthic invertebrates (Casini et al. 2004; Möllmann et al. 2004; Røjbek et al. 2014). Higher proportions of the minor MUFAs 16:1n-7 and 18:1n-7 in herring compared to sprat appear to result from feeding preferences. The contents of these two MUFAs, and more pronouncedly that of 16:1n-7, increase in herring as a function of age (Keinänen et al. 2017), i.e., with increased feeding on benthic invertebrates (Casini et al. 2004; Möllmann et al. 2004). High proportions of 16:1n-7 have similarly been found in other fish species that consume benthic invertebrates (Happel et al. 2018). Comparing salmon from the two areas, the difference was significant among these FAs only for 18:1n-7, i.e., the elongation product of 16:1n-7. Thus, apart from reflecting herring as a dietary component, this also indicates ongoing fatty acid metabolism in salmon.

### An abundant diet rich in lipids and PUFAs results in thiamine deficiency

In the M74 year, the M74 females were larger than non-M74 females. This, together with the decrease in YSFM and increase in the THIAM concentration of eggs as a function of decreasing fish size, suggests, consistently with the FA signatures, that non-M74 females had probably been feeding in the northern areas of the Baltic Sea, e.g., in the BS or in the northern BPr. In these areas, the lipid content in the prey fish biomass of salmon has been lower than in the southern BPr (Keinänen et al. 2012). In the BS, salmon have generally been smaller and their growth rate has been slower than in the southern Baltic Sea (Salminen et al. 1994; Keinänen et al. 2012). In any case, non-M74 females must on average have consumed prey with a lower energy density than M74 females, and on the basis of the FA signature, a greater proportion of the diet comprised herring. Conversely, the FA profile and larger size of the M74 females indicate that they had most probably been feeding in the BPr and in its subareas, where the prey fish biomass has largely consisted of energy-rich fatty sprat (Mikkonen et al. 2011; Keinänen et al. 2012). A high and moderate incidence of M74 has been associated with a large body weight or high CF of pre-spawning or spawning salmon (Karlsson et al. 1999a; Mikkonen et al. 2011). Consistently, a low egg thiamine concentration of salmonids in connection with thiamine deficiency EMS in the Great Lakes of North America was related to a large fish size (Wolgamood et al. 2005; Werner et al. 2006). On the other hand, a high growth rate and large size of salmonids in those lakes were related to feeding on energy-rich alewife (*Alosa pseudoharengus*) (O’Gorman et al. 1987; Ketola et al. 2009).

As the requirement for thiamine increases with an increase in the energy density of food (Woodward 1994), for Baltic salmon, the need is highest when the prey biomass abundantly consists of young sprat (Keinänen et al. 2012). In relation to the lipid content, the youngest sprat and herring also have the lowest thiamine concentration (Keinänen et al. 2012), which may be an indication of the depletion of thiamine in lipid metabolism in the prey fish themselves (Keinänen et al. 2017). However, the large size of M74 and EMS salmonids, whose offspring suffer from thiamine deficiency, verifies that the thiamine concentration in the diet has been high enough to enable proper growth (Morito et al. 1986). Because of the reduction in the thiamine resources of salmon during the spawning migration and fasting for several months (Karlsson et al. 1999b), the supply of thiamine in relation to the high lipid content and high energy density of the dietary biomass has been too low for successful reproduction (Keinänen et al. 2012).

Considering the concentrations of FAs (i.e. the actual amounts) instead of their proportions, sprat also contain the most DHA, as its concentration increases with an increase in the lipid content in fatty fish species (Alvarez et al. 1998; Hemre and Sandnes 1999; Gélineau et al. 2001; Østbye et al. 2011; Keinänen et al. 2017), as described in Additional Fig. 2 for sprat. A similar lipid dependence in the proportion and concentration of PUFAs in herring and sprat has been demonstrated by Røjbek et al. (2014). Consequently, the concentration of DHA is highest in the youngest sprat, whereas the concentration of 18:1n-9, consistently with the thiamine concentration (Keinänen et al. 2012), is highest in sprat that are some years older (Keinänen et al. 2017). Despite 18:1n-9 being the FA typical of sprat, the role of DHA is probably more important as a cause of thiamine deficiency in salmon. This was suggested by Keinänen et al. (2017), who observed a co-occurrence of the lowest thiamine concentration and highest DHA concentration in the youngest sprat and herring, and noted that young sprat contain double the amount of DHA compared to young herring. A higher DHA/EPA ratio in thiamine-deficient salmonid eggs appears to indicate an increased dietary DHA supply (Pickova et al. 1998, 2003) and thus feeding on lipid-rich prey fish specimens, such as sprat (Keinänen et al. 2012) and alewife (Czesny et al. 2009). Consistently, the higher DHA/EPA ratio of muscle in the present study was linked to a lower THIAM concentration in eggs.

In the early- and mid-1990s, when most salmon yolk-sac fry died of thiamine deficiency during several years, the collapse of the cod stock led to many strong successive year classes of sprat in the Baltic Sea, whereby the sprat stock multiplied (Mikkonen et al. 2011; Keinänen et al. 2012). Differences in the muscle FA profiles of female Baltic salmon spawners between the M74 year (1998) and the non-M74 year (2004) or between M74 females and non-M74 females in 1998 verify that the M74 syndrome, at least in the years with a high or moderate incidence of M74, has in most cases been a consequence of feeding abundantly on sprat (Mikkonen et al. 2011; Keinänen et al. 2012). The same conclusion, based on the similarities in the organochlorine profiles between sprat and salmon that ascended the River Simojoki to spawn in comparison to more dissimilar organochlorine profiles between herring and salmon, was earlier reached by Vuorinen et al. (2002). Moreover, results obtained by estimating the feeding area by SIA demonstrated that ARA was present in lower proportions in those salmon that were assigned to the southern parts of the Baltic Sea, although the SIA method could not perfectly segregate the reference salmon of the BS and the BPr (Torniainen et al. 2017). After having been insignificant for 4 years, M74 returned, apparently as a consequence of an exceptionally strong year class of sprat in 2014 in the Baltic Sea (ICES 2018b). Hence, the egg THIAM concentrations of salmon spawners in the autumns of 2015 and 2016 decreased to approximately a half of those in the previous years, and M74 mortality considerably increased among their offspring in 2016 and 2017.

## Conclusions

To conclude, the FA signature of the diet is manifested in the muscle of spawning salmon, despite the extensive lipid metabolism and subsequent FA mobilisation during the pre-spawning fasting and oocyte development. Among the major FAs, the proportions of 16:0 and of SFAs altogether are lower and those of DHA and PUFAs higher in spawning salmon than in feeding salmon caught in the sea. The most sensitive indicators of the main prey fish of Baltic salmon are ARA and the ratio 18:1n-9/ARA, since the lipid of sprat is characterised by 18:1n-9, whereas herring lipid is most clearly characterised by ARA among n-6 PUFAs. Moreover, feeding in the BPr is indicated by a higher proportion of 14:0. The FA signature of M74 females in a year with a moderate M74 incidence reflected that of sprat and BPr salmon principally feeding on sprat, whereas the FA signature of non-M74 females traced that of herring and BS salmon. The suggestion by Mikkonen et al. (2011) and Keinänen et al. (2012) that thiamine deficiency in Baltic salmon offspring in most cases develops as a result of feeding abundantly on young fatty sprat in the BPr was, thus, verified by the FA signature analysis. As Keinänen et al. (2017) suggested, fatty young sprat specimens, when abundantly preyed on by salmon, easily overload them, particularly with highly unsaturated DHA, thereby predisposing them to oxidative stress, which results in the depletion of thiamine.

## Electronic supplementary material

Below is the link to the electronic supplementary material.

Supplementary material 1 (PDF 304 kb)
