# Supplementary material for: Fatty acid signatures connect thiamine deficiency with the diet of the Atlantic salmon (Salmo salar) feeding in the Baltic Sea
Source: Mar Biol. 2018 Oct 1;165(10):161. doi: 10.1007/s00227-018-3418-8 (PMC6182616; doi:10.1007/s00227-018-3418-8)
Supplement: Supplementary file 1 — Supplementary material 1 (PDF 304 kb) [file 227_2018_3418_MOESM1_ESM.pdf]

Fatty acid signatures connect thiamine deficiency with the diet of the Atlantic salmon (*Salmo salar*) feeding in the Baltic Sea

Marja Keinänen[1], Reijo Käkelä[2], Tiina Ritvanen[3], Jukka Pönni[1], Hannu Harjunpää[4], Timo Myllylä[5] and Pekka J. Vuorinen[1]

[1]*Natural Resources Institute Finland (Luke), P.O. Box 2, FI-00791 Helsinki, Finland,* [2]*Department of Biosciences, P.O. Box 65, FI-00014 University of Helsinki, Finland,* [3]*Finnish Food Safety Authority Evira, Mustialankatu 3, FI-00790 Helsinki, Finland,* [4]*Natural Resources Institute Finland (Luke), Puuvillakuja 6, FI-65200 Vaasa, Finland,* [5]*Natural Resources Institute Finland (Luke), Itäinen Pitkäkatu 4 a, FI-20520 Turku, Finland*

Marine Biology

Supplement Table Pearson correlation coefficients (with the *P*-value below) of female-specific offspring mortality during the yolk-sac phase (YSFM) and concentrations of thiamine components (thiamine pyrophosphate, TPP; thiamine monophosphate, TMP; free thiamine, THIAM) and total thiamine (TotTHIAM) in unfertilized eggs with the proportions of muscle fatty acids (FAs) and some of their ratios in 2nd sea-year female salmon spawners (N = 21) from the River Simojoki in 1998, as well as with the total body weight, length and condition factor (CF). A significant (P < *0.1*) correlation between the FA and YSFM and THIAM is indicated by the correlation coefficient being in bold face.

	YSFM	TPP	TMP	THIAM	TotTHIAM
Saturated FAs (SFA)					
14:0	**0.483**	-0.276	-0.589	**-0.563**	-0.583
	0.026	*0.226*	*0.005*	*0.008*	*0.006*
15:0	0.116	0.328	0.190	-0.080	-0.010
	0.617	*0.146*	*0.409*	*0.730*	*0.966*
16:0	-0.003	0.218	-0.076	0.044	0.070
	0.990	*0.343*	*0.744*	*0.849*	*0.764*
17:0	0.207	0.302	0.066	-0.166	-0.099
	0.368	*0.184*	*0.776*	*0.471*	*0.670*
18:0	-0.137	0.344	0.263	0.313	0.350
	0.553	*0.127*	*0.249*	*0.168*	*0.120*
20:0	**-0.398**	-0.226	0.183	**0.462**	0.391
	0.074	*0.325*	*0.428*	*0.035*	*0.080*

	YSFM	TPP	TMP	THIAM	TotTHIAM
SFA	0.171	0.330	-0.116	-0.085	-0.031
	0.458	*0.144*	*0.616*	*0.715*	*0.893*
Monounsaturated FAs (MUFA)					
14:1*n-5*	-0.144	0.157	0.199	0.052	0.082
	0.534	*0.497*	*0.388*	*0.824*	*0.722*
16:1*n-7*	-0.122	0.027	0.250	0.219	0.215
	0.600	*0.907*	*0.275*	*0.341*	*0.349*
17:1*n-8*	0.231	0.389	0.278	-0.185	-0.089
	0.314	*0.082*	*0.222*	*0.423*	*0.700*
18:1*n-9*	0.146	-0.423	-0.445	-0.136	-0.214
	0.527	*0.056*	*0.043*	*0.556*	*0.352*
18:1*n-7*	**-0.379**	0.332	0.510	**0.418**	0.457
	0.090	*0.142*	*0.018*	*0.059*	*0.037*
20:1*n-9*	-0.362	-0.282	0.237	0.332	0.268
	0.107	*0.215*	*0.301*	*0.142*	*0.241*
MUFA	-0.053	-0.306	-0.144	0.097	0.031
	0.818	*0.177*	*0.535*	*0.676*	*0.893*
Polyunsaturated FAs (PUFA)					
18:2*n-6*	-0.175	-0.200	0.376	0.254	0.219
	0.448	*0.384*	*0.093*	*0.266*	*0.341*
20:2*n-6*	-0.306	-0.151	0.422	0.300	0.271
	0.178	*0.514*	*0.057*	*0.186*	*0.235*
18:3*n-3*	-0.104	-0.029	-0.051	-0.003	-0.010
	0.654	*0.902*	*0.827*	*0.989*	*0.964*
20:4*n-6* (ARA)	**-0.479**	0.008	0.307	**0.739**	0.684
	0.028	*0.973*	*0.175*	*0.0001*	*0.001*
20:5*n-3* (EPA)	-0.197	0.009	-0.071	0.298	0.266
	0.393	*0.969*	*0.760*	*0.189*	*0.245*
22:5*n-3* (DPA)	-0.292	-0.230	0.294	0.146	0.112
	0.199	*0.315*	*0.195*	*0.529*	*0.630*
22:6*n-3* (DHA)	-0.086	0.045	-0.225	-0.102	-0.097
	0.710	*0.845*	*0.327*	*0.660*	*0.675*

	YSFM	TPP	TMP	THIAM	TotTHIAM
PUFA	-0.230	-0.030	-0.102	0.082	0.064
	0.316	*0.897*	*0.662*	*0.723*	*0.785*
n-3 PUFA	-0.124	0.033	-0.201	-0.026	-0.029
	0.592	*0.886*	*0.383*	*0.912*	*0.900*
n-6 PUFA	-0.278	-0.178	0.422	**0.370**	0.329
	0.223	*0.441*	*0.057*	*0.099*	*0.145*
FA ratios					
14:0/ARA	**0.610**	-0.087	-0.464	**-0.729**	-0.696
	0.003	*0.707*	*0.034*	*0.0002*	*0.001*
18:1*n-9*/DHA	0.100	-0.246	-0.047	0.017	-0.026
	0.667	*0.282*	*0.841*	*0.941*	*0.912*
n-3 PUFA/*n-6* PUFA	0.080	0.151	-0.291	-0.222	-0.193
	0.732	*0.514*	*0.201*	*0.333*	*0.403*
18:1*n-9*/16:1*n-7*	0.212	-0.399	-0.573	-0.297	-0.362
	0.357	*0.073*	*0.007*	*0.192*	*0.107*
18:1*n-9*/18:1*n-7*	0.192	-0.334	-0.401	-0.215	-0.268
	0.405	*0.139*	*0.072*	*0.350*	*0.239*
18:1*n-9*/18:2*n-6*	0.257	-0.096	-0.649	-0.337	-0.355
	0.261	*0.678*	*0.002*	*0.135*	*0.114*
18:1*n-9*/20:2*n-6*	**0.443**	0.007	-0.597	**-0.493**	-0.477
	0.044	*0.977*	*0.004*	*0.023*	*0.029*
18:1*n-9*/ARA	**0.559**	-0.184	-0.483	**-0.706**	-0.692
	0.008	*0.426*	*0.027*	*0.0003*	*0.001*
18:1*n-9*/n-6 PUFA	**0.387**	-0.110	-0.718	**-0.479**	-0.489
	0.083	*0.636*	*0.0002*	*0.028*	*0.024*
18:1*n-9*/PUFA	0.231	-0.292	-0.285	-0.153	-0.199
	0.314	*0.198*	*0.211*	*0.509*	*0.386*
MUFA/PUFA	0.121	-0.139	-0.017	-0.009	-0.031
	0.601	*0.549*	*0.940*	*0.968*	*0.894*
DHA/EPA	0.125	0.044	-0.219	**-0.444**	-0.405
	0.589	*0.850*	*0.340*	*0.044*	*0.069*

	YSFM	TPP	TMP	THIAM	TotTHIAM
Salmon					
Weight	**0.498**	-0.371	-0.501	**-0.646**	-0.668
	0.022	*0.097*	*0.021*	*0.002*	*0.001*
Length	**0.532**	-0.438	-0.578	**-0.695**	-0.727
	0.013	*0.047*	*0.006*	*0.001*	*0.0002*
CF	0.068	0.061	0.008	-0.204	-0.173
	0.769	*0.794*	*0.972*	*0.376*	*0.453*

Fatty acid signatures connect thiamine deficiency with the diet of the Atlantic salmon (*Salmo salar*) feeding in the Baltic Sea

Marja Keinänen[1], Reijo Käkelä[2], Tiina Ritvanen[3], Jukka Pönni[1], Hannu Harjunpää[4], Timo Myllylä[5] and Pekka J. Vuorinen[1]

[1]*Natural Resources Institute Finland (Luke), P.O. Box 2, FI-00791 Helsinki, Finland,* [2]*Department of Biosciences, P.O. Box 65, FI-00014 University of Helsinki, Finland,* [3]*Finnish Food Safety Authority Evira, Mustialankatu 3, FI-00790 Helsinki, Finland,* [4]*Natural Resources Institute Finland (Luke), Puuvillakuja 6, FI-65200 Vaasa, Finland,* [5]*Natural Resources Institute Finland (Luke), Itäinen Pitkäkatu 4 a, FI-20520 Turku, Finland*

Marine Biology

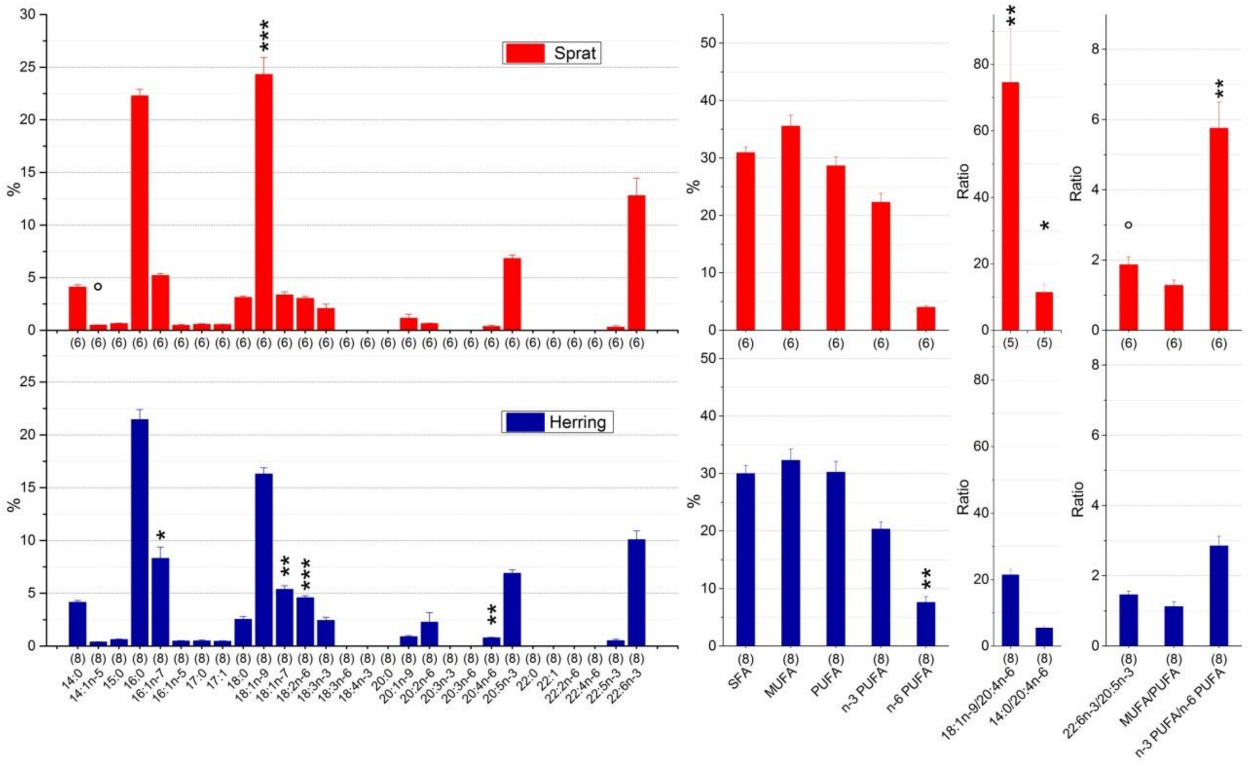

Additional Fig. 1 Profiles of fatty acids (proportions), their sums and some ratios in sprat from the Baltic Proper (BPr) and herring from the Bothnian Sea (BS). Mean (± SE) proportions are given and significant (o = $P < 0.1$, * = $P < 0.05$, ** = $P < 0.01$ and *** = $P < 0.001$) differences between sprat and herring are indicated. Fatty acid data are from Keinänen et al. (2017)

Fatty acid signatures connect thiamine deficiency with the diet of the Atlantic salmon (*Salmo salar*) feeding in the Baltic Sea

Marja Keinänen[1], Reijo Käkelä[2], Tiina Ritvanen[3], Jukka Pönni[1], Hannu Harjunpää[4], Timo Myllylä[5] and Pekka J. Vuorinen[1]

[1]*Natural Resources Institute Finland (Luke), P.O. Box 2, FI-00791 Helsinki, Finland,* [2]*Department of Biosciences, P.O. Box 65, FI-00014 University of Helsinki, Finland,* [3]*Finnish Food Safety Authority Evira, Mustialankatu 3, FI-00790 Helsinki, Finland,* [4]*Natural Resources Institute Finland (Luke), Puuvillakuja 6, FI-65200 Vaasa, Finland,* [5]*Natural Resources Institute Finland (Luke), Itäinen Pitkäkatu 4 a, FI-20520 Turku, Finland*

Marine Biology

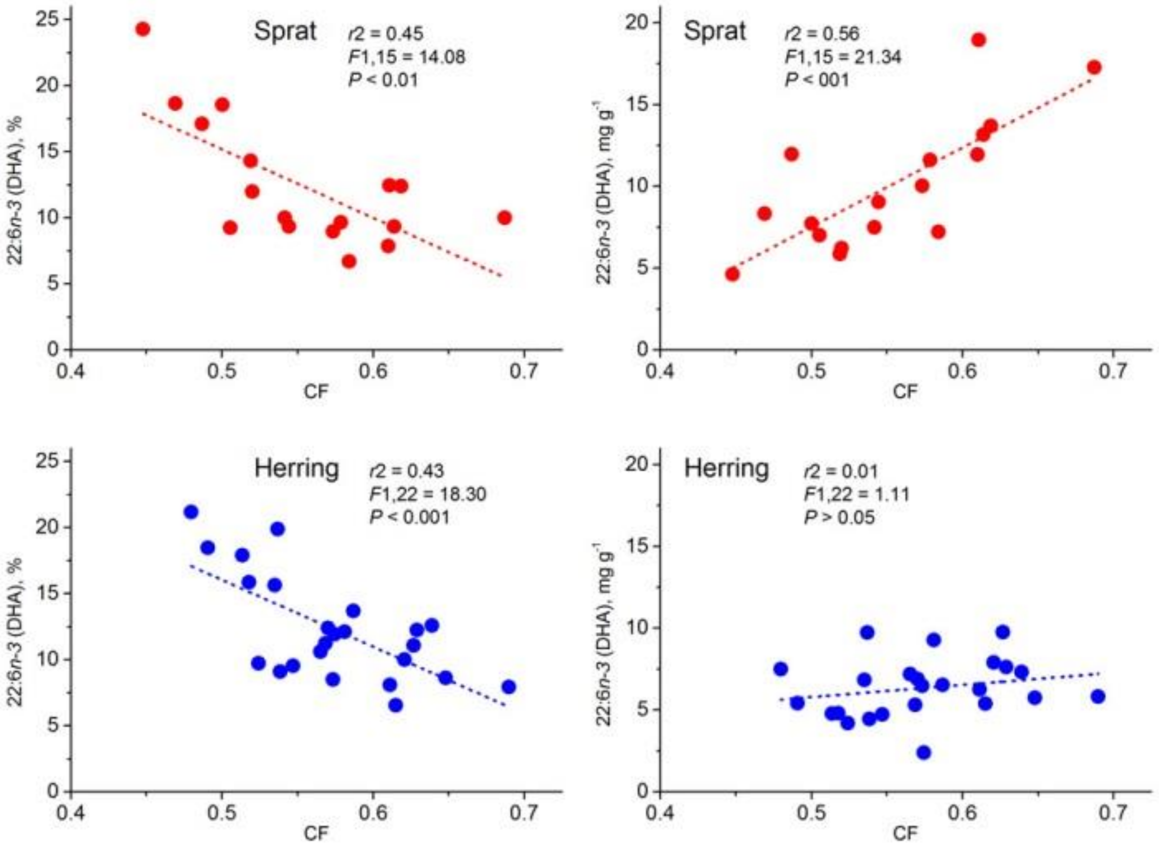

Additional Fig. 2 Proportion (left) and concentration (right) of docosahexaenoic acid (DHA, 22:6*n*-3) in the whole body of salmon prey fish, sprat and herring, in relation to the condition factor (CF). Fatty acid data are from Keinänen et al. (2017)
